# Understanding the Influence of Secondary Metabolites in Plant Invasion Strategies: A Comprehensive Review

**DOI:** 10.3390/plants13223162

**Published:** 2024-11-11

**Authors:** Rasheed Akbar, Jianfan Sun, Yanwen Bo, Wajid Ali Khattak, Amir Abdullah Khan, Cheng Jin, Umar Zeb, Najeeb Ullah, Adeel Abbas, Wei Liu, Xiaoyan Wang, Shah Masaud Khan, Daolin Du

**Affiliations:** 1 Institute of Environment and Ecology, School of Environment and Safety Engineering, Jiangsu University, Zhenjiang 212013, China; rasheed.akbar@uoh.edu.pk (R.A.);; 2 Department of Entomology, Faculty of Physical and Applied Sciences, The University of Haripur, Haripur 22062, Khyber Pakhtunkhwa, Pakistan; 3 Jiangsu Collaborative Innovation Center of Technology and Material of Water Treatment, Suzhou University of Science and Technology, Suzhou 215009, China; 4 College of Life Sciences and Oceanography, Shenzhen University, Shenzhen 518060, China; 5 School of Food and Biological Engineering, Jiangsu University, Zhenjiang 212013, China; 6 Department of Chemical and Biomolecular Engineering, University of Tennessee, Knoxville, TN 37996, USA; 7 College of Optical, Mechanical and Electrical Engineering, Zhejiang A&F University, Hangzhou 311300, China; 8 Department of Horticulture, Faculty of Physical and Applied Sciences, The University of Haripur, Haripur 22062, Khyber Pakhtunkhwa, Pakistan; 9 Jingjiang College, Jiangsu University, Zhenjiang 212013, China

**Keywords:** allelopathy, invasive species, herbivory, integrated weed management, phytochemicals

## Abstract

The invasion of non-native plant species presents a significant ecological challenge worldwide, impacting native ecosystems and biodiversity. These invasive plant species significantly affect the native ecosystem. The threat of invasive plant species having harmful effects on the natural ecosystem is a serious concern. Invasive plant species produce secondary metabolites, which not only help in growth and development but are also essential for the spread of these plant species. This review highlights the important functions of secondary metabolites in plant invasion, particularly their effect on allelopathy, defense system, interaction with micro soil biota, and competitive advantages. Secondary metabolites produced by invasive plant species play an important role by affecting allelopathic interactions and herbivory. They sometimes change the soil chemistry to make a viable condition for their proliferation. The secondary metabolites of invasive plant species inhibit the growth of native plant species by changing the resources available to them. Therefore, it is necessary to understand this complicated interaction between secondary metabolites and plant invasion. This review mainly summarizes all the known secondary metabolites of non-native plant species, emphasizing their significance for integrated weed management and research.

## 1. Introduction

In recent years, along with the development of traffic networks, the increase of human activity and the strengthening of international trade, the interaction among biological species from diverse habitats has significantly increased. Some of these species have shown a high ability to adapt to their new environments, allowing them to flourish and spread quickly. This trend has adversely affected the local economy, ecology, and society [1]. Plant species have been unintentionally or purposefully brought to other continents for ornamental purposes. Due to climate change, species distributions have shifted over the last few decades [2]. Biological invasions are caused by species reorganization and climate change, which allow exotic species to grow progressively in native ecosystems [3]. Plant invasion is a primary threat to natural ecosystems and causes global issues [4]. As plants invade new areas, the introduction of invasive species may have a significant negative impact on local plant species. Decreasing the richness or diversity of native plants potentially leads to limited diversity in numerous portions of the invaded range [5]. In fact, similar ecological effects have been seen in invaded communities by invasive species, including *Centaurea maculosa*, *Solidago canadensis*, *Mikania micrantha*, *Alternanthera philoxeroides*, *Eupatorium odoratum*, and *Fallopia japonica* [6]. However, the specific mechanisms that lead to this dominance remain elusive and require further investigation for clarification [7].

### 1.1. Invasion Mechanism

Several theories exist on the potential for exotic plants to become invasive [8]. Plant invasions are major mechanisms described in the literature, such as:

#### 1.1.1. The Enemy Release Hypothesis

The enemy release hypothesis is known as the predator escape or ecological release. The herbivore escape hypothesis refers to the absence of competitors, predators and pathogens in the introduced range as the main advantage for an introduced species [9]. The absence of predators allows the plant to allocate energy and resources towards competitive traits [10]. The enemy escape hypothesis suggests that: (a) natural enemies play a crucial role in shaping and controlling plant populations; (b) native plant species are generally more susceptible to biocontrol agents than non-native species; and (c) the reduced presence or absence of natural enemies contributes to the accelerated growth and proliferation of exotic species [11]. Based on these assumptions, the main method for controlling exotics was the introduction of biocontrol agents, which has not always been successful [12].

#### 1.1.2. The Novel Weapons Hypothesis

The novel weapons hypothesis suggests that plants use biochemical mechanisms to conquer new environments [13]. Invasive species not only escaped from their natural enemies but also brought novel mechanisms of interaction to invade plant communities [14]. These exotics possessed phytochemicals, unique metabolites, and biochemical mechanisms that recipient communities have not previously encountered. Invasive plant secondary metabolites (chemical weapons) have interfered with native plants and were successfully established in the native range [15]. Nowadays, recent research has shown a diversity of phytochemical differences between native and invasive plant species [14], and these differences can be distinguished by metabolomics and metabolic profiling. Allelopathy is also an important phenomenon in which the invasive plant releases some phytochemicals and retard the growth and development of neighboring plants [16]. Most invasive plant species release allelochemicals (phenolic compounds, alkaloids, and terpheniods) into the nearby environment by litter or root exudation [15]. These phytochemicals influence the growth attributes of nearby plants, such as root growth, germination, and nutrient intake. It is necessary to figure out why invasive plant species spread quickly in an area and how to manage them. We need to know how allelopathy works.

#### 1.1.3. Resistance Against Herbivores

The main factor in the success of invasive plant species is the resistance against herbivores. Invasive plant species use different strategies to compete with native plants and protect themselves from herbivores [17]. These defense mechanisms against herbivores are the production of secondary metabolites (phenolic compounds, terpheniods, and alkaloids) that might be poisonous or repel herbivores [18]. In a new environment, the invasive plant species can decrease the herbivory attack and focus on more resources to grow and reproduce by becoming dominant over the native plant species.

#### 1.1.4. Secondary Metabolites

Invasive plant species utilize various secondary metabolites, which enhance their competitive ability over native plants and help in nutrient uptake. Malic acid and citric acid are the organic acids produced in invasive plant species’ rhizosphere. The function of these compounds is to solubilize nutrients, such as phosphorous, so that it is easy for the plant roots to absorb [19].

#### 1.1.5. Antimicrobial Abilities

Antimicrobial abilities are another mechanism of invasive plant species that enables them to invade successfully [20]. Invasive plant species produce secondary metabolites such as phytoalexins, alkaloids, and essential oils, which are rich in compounds like limonene, pinene, terpinene, and caryophyllene [21]. These metabolites possess antimicrobial properties that alter the microbial ecosystem, creating favorable environmental conditions that support the growth and establishment of invasive plants while inhibiting the growth of native plant species [22].

#### 1.1.6. Mutualistic Interactions

During the invasion process of invasive plants, secondary metabolites played important mutualistic interactions and served different functions. According to [23], flavonoids, such as kaempferol and quercetin, are important signaling molecules that promote mutualistic relationships with mycorrhizal fungi. These flavonoids act as chemo attractants, facilitating the growth and development of mycorrhizal hyphae and enhancing the invasive plant’s nutrition intake. Isoflavonoids, like genistein and daidzein, are produced by leguminous invasive species and play a key signaling role in attracting and forming symbiotic relationships with nitrogen-fixing bacteria in root nodules [24]. The precise role of these secondary metabolites facilitates the beneficial interactions that sustenance the growth of invasive plant species.

In invasive plant species, the production of secondary metabolites is important and gives advantages over native flora, aiding in establishment within new habitats. These phytochemical compounds are used by the invasive plant as a defense against the herbivory. Also, they inhibit the germination, growth, root development, and intake of nutrients of native plant species. Alkaloids and phenolic compounds are examples of these compounds [25]. This enables invasive plant species to invade and grow quickly, making the environment unsuitable for the native plant species. Secondary metabolites from the invasive plant species affect the soil microbial population by preventing beneficial microorganisms and enhancing pathogenic growth [26]. These interruptions in the microbial balance in the soil further strengthen the ability of invasive plant species to control the resources, and the competition they face from native plant species decreases. Invasive plant species produce secondary metabolites that play an important role in determining the ecological interactions within invaded ecosystems, supporting the plant establishment. The generation and discharge of secondary metabolites by invasive plants are crucial in determining the nature of ecological interactions in invaded ecosystems, which, in turn, help these plants establish and proliferate successfully.

Investigating the mechanisms and functions of phytochemical compounds in the invasion of invasive plant species is an important and significant field, highlighting a notable research gap. Much research has provided valuable insights into specific functions, like defense mechanisms, stress tolerance, and allelopathy [27]. It has also been reported that secondary metabolites in the invasive plant species strengthen the plant against herbivory and infection [28]. There is a clear gap in the literature where a comprehensive review combines and explores all these results across various invasive plant species. A comprehensive review is necessary to scientifically evaluate the diversity of secondary metabolites and their complex roles in assisting invasion success. A comprehensive review designed within an integrated framework would reveal both shared and different elements among invasive species, contributing to more integrated interpretations of secondary metabolites’ roles in plant invasion dynamics. The current literature commonly focuses on the biochemical roles of secondary metabolites, but there is a scientific gap about the physiological and essential molecular mechanisms regulating these roles. A comprehensive review should not only collect the existing research studies but also focus on the functions of these aspects. This will guide future research and provide a better understanding of the role of secondary metabolites in plant invasions. Filling this gap will greatly improve our knowledge and help construct effective management strategies.

## 2. Secondary Metabolites in Invasive Plants

Plants released secondary metabolites, which may have affected the growth and development of nearby plant species. When organic matter breaks down in plants, secondary metabolites are produced and released, which may be reached by precipitation and influence interactions between soil microbes and plants [29]. Phytochemical compounds may be divided into defense compounds and signal substances [30]. Signal substances facilitate allelobiosis, while defense compounds, i.e., allelochemicals, facilitate allelopathy. A maximum of the secondary metabolites is made up of various organic compounds, which may be volatile or non-volatile. The phytochemicals include alkaloids, glycosides, organic acids, phenols, and terpeniods [30]; these compounds are important for regulating the rhizosphere microbiota, proper plant growth, and defense systems [31]. It is essential to understand what makes a plant invasion successful [8,32]. Research in the past showed that to improve their effectiveness, invasive plants produce secondary metabolites [33]. These secondary metabolites have influenced the cycling of nutrients, native plants, and soil microbe [34]. To mediate these effects, both direct and indirect routes can be used. Observing how chemicals from invasive plants affect plant competition—by limiting the growth of nearby plants—provides a straightforward way to understand their effects [35]. According to [36], the growth of the native plants is inhibited immediately by the chemical phytotoxin (2)-catechin emitted from the roots of the invading *Centaurea maculosa*. Also, the seeds of native plants are prevented from germination due to the secondary metabolites produced by invaded invasive plant species [37]. It is clear that the increase in competitiveness of the invasive plant may be due to the inhibition of these direct impacts [34]. Invasive plant species release some phytochemicals, such as alkaloids, phenols, terpeniods, and volatile compounds, which indirectly affect the competition. By changing the cycling nutrients—specifically, soil inorganic and organic nutrients flows and pools—these metabolites might unintentionally promote the growth of invaders [38]. Additionally, invasive plants may compete indirectly by using natural soil microbe. Plant pathogens and symbionts, along with secondary metabolites, have the ability to change the soil’s microbial community [39]. These microbes ensure the survival of plants in challenging environments [40]. The reason behind the success of invasive plant invasion over the native plant is the presence of secondary metabolites. Secondary compounds include many substances like growth inhibitors, poisons, and allelopathic chemicals [41]. The following are several secondary metabolites that are mostly present in invasive plants and can harm native plant species.

### 2.1. Phenolic Compounds

**Figure 1:**
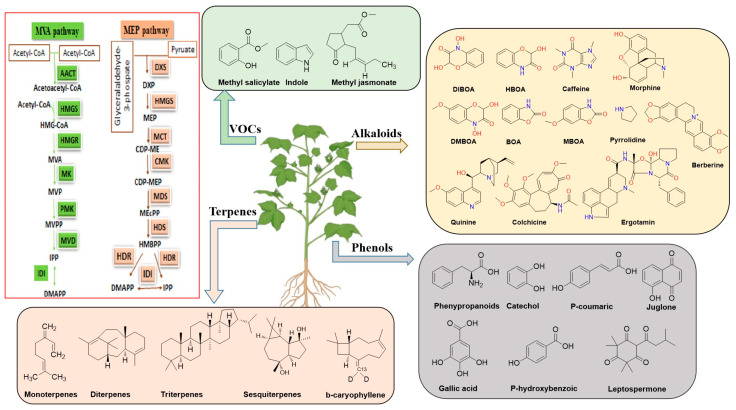
Secondary metabolites in invasive weeds, for the synthesis of isopentenyl diphosphate (IPP), dimethylallyl diphosphate (DMAPP), methylerythritol phosphate (MEP), and mevalonate (MVA) pathways responsible. Acetoacetyl-CoA, 3-hydroxy-3-methylglutaryl-CoA (HMG-CoA), acetyl-CoA, acetoacetyl-CoA, acetyl-CoA, MVA, 5-phosphomevalonate (MVP), and 5-diphosphomevalonate (MVPP) are the intermediaries of the MVA pathway. Acetyl-CoA acetyltransferase (AACT), 3-hydroxy-3-methylglutaryl-CoA synthase (HMGS), mevalonate kinase (MK), 3-hydroxy-3-methyl-glutaryl-CoA reductase (HMGR), phosphomevalonate kinase (PMK), diphosphomevalonate decarboxylase (MVD), and isopentenyl diphosphate isomerase (IDI) are the enzymes involved in the MVA pathway. Relatively to the MEP pathway, its intermediaries are D-glyceraldehyde 3-phosphate (G3P), pyruvate, 1-deoxy-d-xylulose 5-phosphate (DXP), MEP, 4-(cytidine 5′-diphospho)-2-C-methyl-D-erythritol (CDP-ME), 2-phospho-4-(cytidine 5′-diphospho)-2-C-methyl-D-erythritol (CDP-MEP), 2-C-methyl-D-erythritol-2,4-cyclodiphosphate (MEcPP), and 1-hydroxy-2-methyl-2-butenyl 4-diphosphate (HMBPP). The enzymes involved in the MEP pathway are 1-deoxy-d-xylulose-5-phosphate synthase (DXS), 1-deoxy-d-xylulose-5-phosphate reductoisomerase (DXR), 2-C-methyl-D-erythritol 4-phosphate cytidylyltransferase (MCT), 4-diphosphocyt-idyl-2-C-methyl-D-erythritol kinase (CMK), 2-C-methyl-D-erythritol 2,4-cyclodi-phosphate synthase (MDS), 4-hydroxy-3-methylbut-2-enyl-diphosphate synthase (HDS), and 4-hydroxy-3-methylbut-2-enyl diphosphate reductase (HDR).

Among the phenolic compounds are tannins, phenolic acids, flavonoids, and coumarins (Figure 1). When present in high concentrations, they can prevent seeds’ α-amylase from working, which reduces germination by 18% [14]. Phenolics, which are oxygen radical producers and feeding deterrents, can have adverse effects. It has been discovered that the creation of hydrogen or covalent connections between phenolic chemicals in herbivores’ intestinal tracts and food proteins or digestive enzymes inhibits digestion [42]. Fall webworm (*Hyphantria cunea* Drury) digestion and food consumption can be greatly impacted by tannic acid [43]. According to [44], flavonoids not only prevent weed growth and fungal pathogen spore germination but also serve as chemical cues for legumes to modulate, and the root exudates of barley (*Hordeum vulgare* L. cv. ‘Barke’) contain phenylpropanoids that have antifungal properties. When discharged in high quantities, phenolic compounds can have negative impacts on animals, soil, and groundwater, even if low concentrations of the same chemicals may discourage pests, prevent infections, or promote beneficial species [45].

Eucalyptus species, such as *E. microtheca*, *E. polycarpa*, *E. tereticornis*, and *E. camaldulensis*, have been found to contain some of the possible phenolic allelochemicals in new leaves, bark, and leaf litter leachates. Studies revealed the existence of catechol, p-coumaric, gallic, and p-hydroxybenzoic, which have detrimental effects on crops in the ecosystem, such as black gram (*Phaseolus mungo* L.), by reducing and delaying germination, seedling mortality, and growth and yield reduction [46]. Black walnuts (*Juglans nigra* L.) produce a phenolic chemical called juglone (5-hydroxy-1,4-naphthalenedione) (Figure 1). It is widely recognized to have a detrimental effect on the growth of other plants. The primary enzyme in the manufacture of plastoquinone, hydroxyphenylpyruvate dioxygenase (HPPD), is strongly inhibited by juglone. It also affects the respiratory and photosynthetic electron transport systems [47]. The natural triketone leptospermone (1-hydroxy-2-isovaloryl-4,4,6,6-tetramethyl cyclohexen-3,5-dione) (Figure 1), produced by the roots of the bottlebrush (*Callistemon citrinus* Curtis), inhibits p-hydroxyphenylpyruvate dioxygenase, contributing to its herbicidal properties. This inhibition causes chlorophyll loss and disrupts carotenoid production. Because of its strong herbicidal effects, commercial development of leptospermone is not possible. Nonetheless, the structure of leptospermone served as a foundation for the creation of synthetic analogs that were utilized to suppress broadleaved weeds in maize. For example, [46] highlight manuka oil, which contains leptospermone as its main active ingredient, can enhance the herbicidal effects of other essential oils. Redroot pigweed, barnyard grass, velvet leaf, and hairy crabgrass showed markedly reduced growth and dry weight when manuka oil (1%) was sprayed on them after they emerged. This type of application offers an additional avenue for using this allelopathic molecule without modifying its chemical structure [48].

### 2.2. Alkaloids

Alkaloids are vital secondary chemicals in plants with significant physiological and biological effects. Citronella (*Cymbopogon nardus* (L.) Rendle) root extract includes N-octanoyl tyramine, which can prevent Italian ryegrass (*Lolium multiflorum* Lam.), lettuce (*Lactuca sativa* L.), barnyard grass (*Echinochloa crus-galli* L.), and cress (*Lepidium sativum* L.) from maturing [49]. Caffeine (1,3,7-trimethixanthine) is a purine alkaloid found in about 100 plant species. It can be utilized directly as an allelopathic toxin or indirectly as an activator of plant defense systems. It can also strengthen a plant’s capacity for defense. Additionally, caffeine can be excreted actively through primary roots, influencing nearby microbial populations [50]. *Echium plantagineum* L. produces harmful pyrrolidine alkaloids to defend against herbivores; naphthoquinone has an effect on insects and livestock and can reduce competition among weeds, insects, and pathogens [51]. Several gramineous species release benzoxazinoids into the rhizosphere to change the bacterial and fungal populations linked with roots and inhibit nearby plants’ growth [52]. Moreover, gramineous species’ roots may release benzoxazinoids to prevent nearby plants from growing [53].

Still, it has been established that certain alkaloids, such as quinine, colchicine, morphine, berberine, ergotamine, and allyl isothiocyanate, demonstrate phytotoxicity and prevent neighboring plants’ seeds from germinating or growing into seedlings. An investigation was carried out in which the alkaloid fraction of *Crotalaria retusa* was gathered and examined for *Phaseolus vulgaris* allelopathic potential at different concentrations. Allelochemicals caused oxidative stress and prevented bean seed germination as concentrations increased [54]. Their phytotoxicity and allelopathic efficacy on weeds have been extensively investigated. Cereals create a variety of benzoxazinoids and hydroxamic acids, which are then exuded into the surrounding soil solution from plant tissues and residues during decomposition and root exudation (from root hairs or secondary roots). These compounds include benzoxazolin-2(3H)-one (BOA), benzoxazinones 2,4-dihydroxy-7-methoxy-(2H)-1,4-benzoxazin-3(4H)-one (DIMBOA), 2-hydroxy7-methoxy-1,4-benzoxazin-3-one (HMBOA), 2-hydroxy-1,4-benzoxazin-3-one (HBOA), 6-methoxy-benzoxazolin-2-one (MBOA), and 2,4-dihydroxy-(2H)-1,4-benzoxazin-3(4H)-one (DIBOA (Figure 1) [52]. It has been demonstrated that they exhibit physicochemical and microbiological changes after release, which results in modifications to phytotoxicity mediated by microbes [55]. When external stimuli are released into the cytoplasm, benzoxazinones are retained in vacuoles in the glucosidic form, where they are digested by β-glucosidases to increase their reactivity and biological activity [56]. While the benzoxazolinone breakdown products, MBOA and BOA, are thought to be less bioactive than the initial molecules, the unstable benzoxazinone aglucones, DIBOA and DIMBOA, are poisonous. Nevertheless, research has demonstrated that the glucosides of DIBOA and DIMBOA, along with their corresponding aglycones and degradation products, control weeds such as redroot pigweed, barnyard grass, and crabgrass [57]. Alkaloids, such as pyrrolizidine alkaloids in *Chromolaena odorata*, act as deterrents to herbivores and other natural enemies, reducing the impact of predation and facilitating the plant’s establishment in new environments [58]. Also, these compounds exhibit allelopathic properties, inhibiting the germination and growth of surrounding plant species, which helps invasive plants like *Chromolaena* and *E. adenophorum* dominate native flora [59]. In essence, alkaloids and other allelopathic substances give invasive species a competitive edge by suppressing native plants and enhancing their resilience against herbivory and other environmental stresses, contributing to their successful spread and establishment [60]. Understanding the specific mechanisms and concentrations of these compounds in the environment is crucial for developing strategies to manage invasive plant species effectively

### 2.3. Terpenes

Terpenoids are vital substances found in nature that are divided into four groups according to the number of isoprene units in their carbon structure: monoterpenes, sesquiterpenes, diterpenes, and triterpenes [61]. These two distinct mechanisms produce isopentenyl diphosphate (IDP) and dimethylallyl diphosphate (DMADP), essential for the synthesis of terpenoid compounds. Figure 1 shows the pathways for mevalonic acid in the cytosol, endoplasmic reticulum, peroxisomes, and methylerythritol phosphate (MEP) in plastids. Learning these complex systems and how they function in biological processes helps us better understand how these compounds are made [62]. Terpeniods function not only as allelochemicals and reproductive hormones but also provide photoprotection, which is vital to plants because they mediate polysaccharide assembly. The literature has highlighted the inhibitory effect of seedling germination and growth, exhibiting an autotoxic and allelopathic nature. These changes are the results of complex interactions relating ATP (Adenosine triphosphate) production alteration, endocrine activity, protein complexation, and respiratory blockage. According to [46], terpenoids are important not only for plants’ environment but also for defense and communication. Terpeniods have tremendous characteristics that help invasive plants attract pollinators and protect the plant from herbivores and microbes. A lot of research work was conducted on how certain chemicals from invasive plants can affect other plants, revealing compelling results. There is one chemical called β-caryophyllene, which is mainly found in plant aromas. It can hinder the seeds from growing into plants in *Brassica napus* L. and *Raphanus sativus* L. [63]. Researchers have thoroughly examined how certain plant compounds, i.e., monoterpenes and sesquiterpenes, have phytotoxic effects against other plant species. In many cases, invasive species produce higher concentrations of terpenes, which can deter generalist herbivores commonly found in new environments, making the invaders less vulnerable to predation compared to native plants [64]. Terpenes can have allelopathic effects, releasing chemicals into the soil to inhibit the growth of neighboring plants. This can significantly alter the composition of native plant communities, providing invasive species with more access to critical resources like nutrients, light, and space [65]. Beyond their defensive role, terpenes also enable invasive species to adapt to a range of abiotic stresses, such as temperature extremes, drought, and salinity. These compounds help maintain the plant physiological functions under challenging conditions, allowing them to thrive in diverse environments where native species may struggle [25]. Many invasive plants adopt a strategy of producing low-cost chemical defenses, like terpenes, which allows them to allocate more energy toward growth and reproduction. This balance between rapid growth and effective chemical defense often results in higher reproductive success and faster spread of invasive species in new habitats [66]. The ecological impact of terpenes extends beyond direct competition, as their presence can modify interactions within the ecosystem, influencing pollinators, herbivores, and soil microbial communities. These alterations can lead to shifts in biodiversity and ecosystem function, often disadvantaging native species. Understanding the role of terpenes in plant invasions is thus essential for developing strategies to manage invasive populations and protect native ecosystems [67]. By shedding light on these biochemical mechanisms, research can inform more effective management practices aimed at mitigating the ecological impacts of invasive species.

### 2.4. Volatile Organic Compounds

The compounds in the air are volatile organic compounds (VOCs), which may be ethylene, methyl jasmonate, methyl salicylate, and indole (Figure 1). These VOCs help the plant species interact with the environment comprising other plant species, herbivores, natural enemies, pollinators, and microbes [68]. Ethylene activates genes through air diffusion, which helps plants defend themselves. In tobacco (*Nicotiana tabacum* L.), small quantities of ethylene can cause characteristics associated with shade avoidance [69]. According to [70], methyl jasmonate in *Artemisia tridentate* activates the defense genes, as shown in Table 1. Moreover, defense genes can be activated by methyl jasmonate via spreading through the environment [71]. An excellent way to attract the green lacewing (*Chrysopa nigricornis* Burmeister) is with methyl salicylate [72]. Indole acetic acid functions as a quick and efficient aerial priming agent to prime neighboring plants’ tissues for defense [53].

### 2.5. Phytochemicals Reported in Invasion Mechanisms

**Figure 2:**
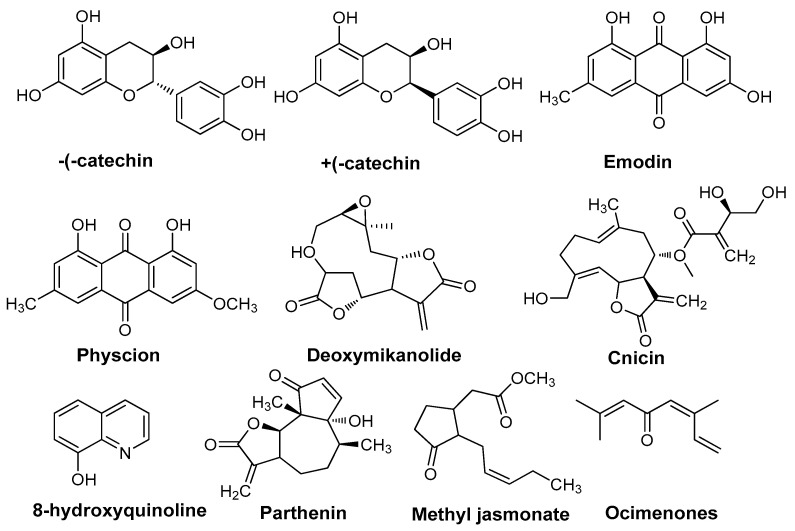
The figure shows a range of secondary metabolites like (−)-catechin, (+)-catechin, cnicin, ocimenones, 8-hydroxyquinoline, deoxymikanolide, emodin, methyl jasmonate, physcion, and parthenin. These compounds are known to play significant roles in plant invasion strategies through allelopathic interactions, where they inhibit native plant growth, disrupt beneficial mycorrhizal fungi associations, and alter the microbial dynamics in the soil. For example, catechins released by invasive species can suppress native vegetation, while methyl jasmonate and emodin may influence plant defense mechanisms and stress responses, enhancing the competitive ability of invasive plants. These biochemical strategies give invasive species a significant ecological advantage.

One of the most economically damaging alien invaders in North America is the *Centaurea* species, which is suspected of displacing native species Catechins quickly through allelopathic mechanisms [77]. (−)-catechin (Figure 2) from *C. maculosa* (Asteraceae), often known as spotted knapweed, was found to be a phytotoxic root exudate, while (+)-catechin (Figure 2) exhibited antibacterial qualities. The idea that *C. maculosa* invasiveness is enabled by (−)-catechin release is supported by the abundance of racemic catechin found in soil extracts from fields where the plant has invaded. In soils sustaining invasive *C. maculosa* in North America, the natural content of (−)-catechin was more than double that in Europe. The findings offer compelling evidence that *C. maculosa* root exudation of (−)-catechin is responsible for the displacement of native plant communities, at least partially. Additionally, they used an integration of ecological, physiological, biochemical, cellular, and genetic techniques to show the allelopathic effects of *C. maculosa*. The findings demonstrated that natural field soil conditions inhibited the growth and germination of native species [78]. The natural content of 8-hydroxyquinoline (8HQ, Figure 2), an allelochemical that has never before been described as a natural product, varies biogeographically from *C. diffusa* (Asteraceae) root exudates [79]. It has larger phytotoxic effects on North American grass species than on Eurasian grass species, and it is at least three times more concentrated in soils invaded by *C. diffusa* in North America than in the native Eurasian soils of this plant. Moreover, regardless of the biogeographical origin of the soil biota, experimental communities constructed from North American plant species are considerably more vulnerable to invasion by *C. diffusa* than communities constructed from Eurasian species. More so than Eurasian soils, North American soil biota sterilization inhibited *C. diffusa*, suggesting that the latter may be encouraged to invade North American soils. Since North American plants have not developed a natural resistance to 8-HQ, it is possible that Eurasian plants and soil microbes have. This suggests a remarkable potential for evolutionary compatibility and homeostasis among plants within natural communities, as well as a mechanism by which exotic weeds ruin these communities. *C. maculosa* (Asteraceae) was shown to have a phytotoxic chemical called cnicin (Figure 2), a sesquiterpene lactone (spotted knapweed) [80]. It can hinder larval growth and development [81]. *M. micrantha* (Asteraceae), commonly known as Mile-a-Minute, is one of the top 100 worst invasive alien species in the world. Deoxymikanolide (Figure 2) and other sesquiterpene lactones were identified from this plant [82], revealing a high level of phytotoxicity to the family Brassica (Brassicaceae). These compounds might significantly impact how well the weed invades [83]. Throughout the plant’s life cycle in a natural population, ocimenones (Figure 2), the predominant terpenes in the essential oils of the leaves and reproductive structures of *Tagetes minuta* L. (Asteraceae) were investigated. Ocimenones’ phytotoxic impact on germination was assessed. According to bioassays, *T. minuta* fruit material and pure ocimenones slowed and prevented coexisting species from germinating. Regarding *T. minuta’s* chemical ecology, a connection between allelopathy, biosynthesis, catabolism, and terpene release is suggested [84]. In field conditions, it was discovered that methyl jasmonate (Figure 2), a trace amount exuded by sagebrush (*Artemisia tridentate* ssp. tridentate, Asteraceae), inhibited *Nicotiana attenuata* seed germination [85,86]. Anthraquinones: in a recirculating system, the root exudates of *Polygonum sachalinense* F. Schmidt ex Maxim. (Polygonaceae) considerably slowed down the growth of lettuce seedlings. Emodin (Figure 2) and physcion’s (Figure 2) inhibitory effects on the growth of seedlings of various tested plant species were demonstrated using TLC agar plates [87]. Emodin and physcion were present in the rhizome, roots, and fallen leaves in comparatively high proportions. This plant community’s soil also contains emodin, with autumnal effective concentrations being the highest. These powerful allelopathic compounds, known as anthraquinones, are therefore likely responsible for the interference seen [16]. The plant parthenium yields a wide range of allelochemicals, which can be classified into many chemical classes. A thorough description of these kinds of allelochemicals emitted by parthenium weed and its residues was provided by [88]. Perthenin (Figure 2) is a sesquiterpene lactone secondary metabolite released from parthenium plants, and this compound affects other plant species [89]. Besides perthenin, parthenium plants also produced a variety of hydrophilic phenols comprising ferulic, anicic, fumaric, vanillic, and caffeic acids. These phenolic compounds exhibit phytotoxicity in water extracts from parthenium species [90]. According to [91], several additional sesquiterpene lactones, flavonoids, and tannins are possible allelochemicals produced by parthenium plant species.

## 3. Functions of Secondary Metabolites

### 3.1. Allelopathy of Invasive Plants

Allelopathy is the chemical exchange of allelochemicals between recipient and donor plants. Certain plant components produce allelochemicals, which are then released into the soil around donor plants, including their rhizosphere, through a variety of mechanisms such as root exudation, rainfall leachates, volatilization of plant parts, or plant residue decomposition [92]. Plant allelopathy has significantly impacted how scarce resources are used and how competition for them exists. The chemical makeup, mechanism of action, and effects of plant root exudates differ, and plants appear to be largely resistant to the allelochemicals they generate. Aqueous extracts from the above-ground portions of *S. canadensis* inhibited the development and germination of *Digitaria sanguinalis* (L.), *Amaranthus retroflexus* L., and *Lactuca sativa* L. [93]. The germination and growth of *Zoysia japonica* (Steud) were suppressed by water-based extracts of *S. canadensis* roots and above-ground parts, while the above-ground parts’ extracts markedly increased malondialdehyde and peroxidase activity [94]. *Raphanus sativus* L. germination was also postponed, and growth was inhibited by preparations of Japanese and Bohemian knotweed using aqueous rhizome. *R. sativus* roots exhibited signs of oxidative stress, including aberrant nuclear, plasma membrane, mitochondrial, and endoplasmic reticulum shapes [95]. The finding suggests that some allelochemicals may infiltrate the seeds and prevent the germination and growth of the seeds. Aqueous extracts of *C. odorata* inhibit the germination of *Ageratum conyzoides* L., *Crassocephalum crepidioides* (Benth.) *S. Moore*, and *Cynodon dactylon* L. [96], and the growth of *Eleusine indica* (L.) Gaertn., *Cyperus iria* L., and *Ageratum conyzoides* L. [97]. According to [98], the growth and germinations of *Amaranthus viridis* L. and *Echinochloa crus-galli* (L.) P. Beauv was inhibited when exposed to aqueous extracts of *C. odorata*. Also, the plant height, leaf area, root length, and plant masses of *Amaranthus spinosus* L. and *Amaranthus spinosus* were decreased when methanol extract of *C. odorata* was sprayed on them [99].

Methanolic extracts of *Mimosa pigra* used against *Ruellia tuberosa*, *Echinochloa crus-galli* (L.) P. Beauv., and *Lactuca sativa* L. affects their growth and development. *M. pigra* extracts are concentration dependent; they interrupt the root mitosis and decrease their cell viability. The allelochemicals from *M. pigra* prevented the native plants from regenerating in the area where these invasive plant species spread [100]. *P. hysterophorus* extract inhibited the germination and growth of *Cyperus iria* L. and showed the same toxic potency as the glyphosate and glufosinate-ammonium synthetic insecticides [101]. Parthenium leaf extracts and residues inhibit the early seedling development and germination of *Phalaris minor* Retz and wild oats *(Avena fatua* L.) in the Petri dish and soil bioassays [102]. The seedling growth and germination of seeds in *Oryza sativa*, *Raphanus sativus*, and *Triticum aestivum* were also delayed by aqueous leaf extract of *M. micrantha* [103]. Aqueous leachates of *M. micrantha* exhibited allelophatic effect against *Aphanus sativus*, *Lactuca sativa*, *Trifolium repens*, and *Lolium multiforum* [104]. The allelochemicals found in *M. micrantha* leaf extracts include vanillic acid, resorcinol, caffeic acid, and p-hydroxybenzaldehyde [105]. *Biden pilosa* L. and *L. perenne* were likewise inhibited from germinating when applying aqueous extracts of *Pueraria montana’s* litter. *B. pilosa* and *L. perenne’s* root and shoot growth was suppressed by the mixture of pure soil and *P. montana* extracts. When compared to the non-infested soil, in the soil infested with *P. montana*, the total phenolic concentration was 30- to 50-fold greater [106]. The results of the experiments point to the possibility that these phenolics are responsible for the inhibition brought on by *P. montana* soils and litter is a new agrochemical tool that has gained recognition for its ability to manage weeds. Growth of *Amaranthus caudatus*, *Amaranthus spinosus*, *Digitaria sanguinalis*, *Lactuca sativa*, *Echinochloa crus-galli*, and *Monochoria vaginalis* was inhibited by plant extracts [107]. When intercropped in citrus orchards, *A. conyzoides* greatly suppressed weeds, including *Cyperus difformis*, *B. pilosa*, and *Digitaria sanguinalis,* [108]. Table 2 summarizes the biological properties of invasive plant species and their allelopathic effect.

#### 3.1.1. Plants Interspecific Allelochemicals

Utilization and competition for scarce resources have been significantly impacted by plant allelopathy and allelobiosis. The chemical makeup, mechanism of action, and effects of plant root exudates differ, and plants appear to be comparatively resistant to the allelochemicals they generate. Furthermore, certain non-allelopathic plants are resistant to the allelochemicals that allelopathic plants produce. The allelochemicals of spotted knapweed (*Centaurea maculosa* Lam.) do not harm eight of the twenty-three grassland species as much as the plant itself does [53]. Strong allelochemicals secreted by certain Asteraceae species can be utilized as “novel weapons” to encroach on new environments, such as *Parthenium hysterophorus* L. [125], *Ambrosia trifida* L. [126], and *C. diffusa* Lam. [127]. However, plants can also use their root exudates to decrease the allelopathy of other plants. *Cunninghamia lanceolata* (Lamb.) Hook., for example, inhibits its growth by releasing cyclic dipeptides (6-hydroxy-1,3-dimethyl-8-nonadecyl-[1,4]-diazocane2,5-diketone) into the soil [128]. By reducing these cyclic dipeptides and lowering their autotoxicity, *M. macclurei* provides chemical signals in a mixed system of *C. lanceolata* and *Michelia macclurei* [129]. The parasite *Striga asiatica* (L.) O. Kuntze’s seeds can undergo allelobiosis for years without finding a host plant. *S. asiatica* parasitizes a host when it recognizes strigolactone released by the host’s roots [130].

The development of two Asian original plant species, namely *Gnaphalium affine* D. Don and *Xanthium sibiricum* Patrin ex Widder, as well as two tropical species, *Aster subulatus* Michx. and *Sesbania cannabina* (Renz.) Poir., and a cosmopolitan species, *Eclipta prostrata* (L.), was significantly suppressed by root exudates of *S. canadensis* gathered from its aeroponic culture. Moreover, *Arabidopsis thaliana* (L.) Heynh’s growth was inhibited by *S. canadensis* root exudates [131]. These data indicated that some allelochemicals that may restrict growth would be released into the rhizosphere soil as *S. canadensis* root exudates and that the amount of these released allelochemicals may be higher in invading ranges than in native ranges. It is well-recognized that *Ageratina adenophora* negatively affects natural vegetation [132]. It influences species diversity, abundance, and the composition of plant communities. This plant is responsible for the decline in the diversity of native species in Nepal’s overrun areas [133]. Allelopathy is one of the ways that *A. adenophora* affects other plants. *A. adenophora’s* allelopathy occurred by leachates; three compounds were isolated and determined to be the primary allelochemicals: 6-hydroxy-5-isopropyl-3, 8-dimethyl-4a, 5, 6, 7, 8, and 8a-hexahydronaphthalen2(1H)-one (HHO), 4,7-dimethyl-1-(propan-2-ylidene)-1, 4,4a, and 8a tetrahydronaphthalene-2, 6(1H, 7H)-dione (DTD) [134,135].

Under field conditions, bohemian knotweed decreased the growth and survival rate of native plants, such as *Acer saccharinum* L. and *Eupatorium perfoliatum* L. Although the knotweed’s inhibitory effects were partially reversed, only a small amount of the native plants’ development conditions were improved with additional nutrients and light. Thus, bohemian knotweed’s allelopathy may partially account for the decline in the growth and survival rate of native plant species [136]. *Centaurea stoebe* L., an invasive plant species, was thought to use (−)-catechin as an allelochemical to continue its invasion of North America because its inhibitory activity was greater than that of (+)-catechin. According to their theory [137], this substance may be released into the soil from the roots of *C. stoebe*, inhibiting the germination and growth of native plant species and disrupting their regeneration. Nonetheless, considerably less catechin was discovered in the field soil to prevent the establishment of local plant species [138]. *Cuscuta chinensis* Lam. seedlings have the ability to discriminate between volatile compounds emitted by the host tomato and non-host wheat. They also grow more selectively toward the tomato plant and successfully parasitize it [53].

#### 3.1.2. Plants Intraspecific Allelochemicals

**Figure 3:**
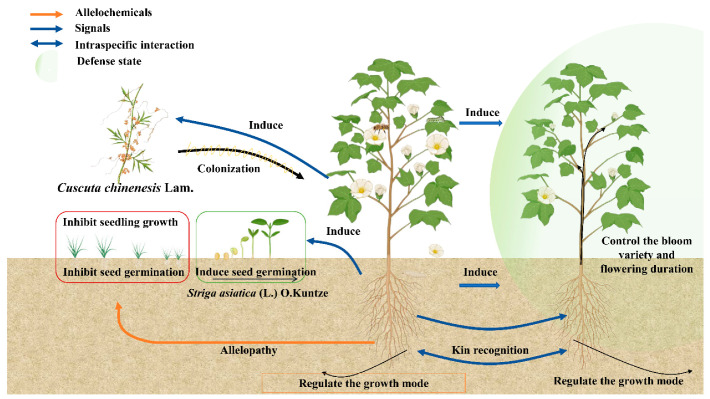
Shows the roles of allelopathy and allelobiosis in plant interactions, focusing on interspecific and intraspecific relationships. Allelopathy, represented by chemical signals, affects the growth of nearby plants, either inhibiting or promoting seed germination. This interaction influences interspecific dynamics, as seen in the impact of *Cuscuta chinensis* on different host species. Allelobiosis involves signaling between plants, including kin recognition, which allows intraspecific regulation of growth and adaptation. Together, these processes shape how plants, both within the same species and among different species, adapt to parasitic pressures and competition.

Plants of the same species have the ability to poison surrounding individuals by producing autotoxic allelochemicals [53]. This phenomenon inside the natural ecosystem is self-thinning. Furthermore, plants have the ability to suppress their own seed germination and seedling growth in order to control the population in both space and time. This allows them to avoid internal competition and increase their geographic range [139]. Autotoxicity is also found in agroecosystems [140] and medicinal plants [141], resulting in a drop in output, inadequate seedling growth, and lower-quality leaves. Chinese fir roots release autotoxic compounds that inhibit the plant’s ability to regenerate [43]. Kin recognition is the ability of plants to recognize and react to their neighbors thanks to intraspecific allelobiosis [142], as shown in Figure 3. Plants identify their relations by subterranean chemical signals, which helps them control community competitiveness and growth [143]. According to evolutionary theory, kin selection will favor individuals with the same genes, providing a higher chance of survival in a changing environment when relatives are recognized [144]. Furthermore, to ensure that outcrossing plants can effectively complete pollination, *Brassica para* L. var *nipposinica* (L. H. Bailey) Hanelt’s root exudates can control both aboveground flowering time and flowering duration [53]. Chemical signals from plants that evaporate into the atmosphere also trigger chemical defense responses in nearby plants or the evaporating plants themselves, regulating population density [145]. Higher levels of tetradecane are released by *Holotrichia parallela*-infested maize roots. In order to create protective jasmonic acid and BX in the roots of maize plants, Motschulsky sends a chemical signal to nearby uninfected plants [146].

**Figure 4:**
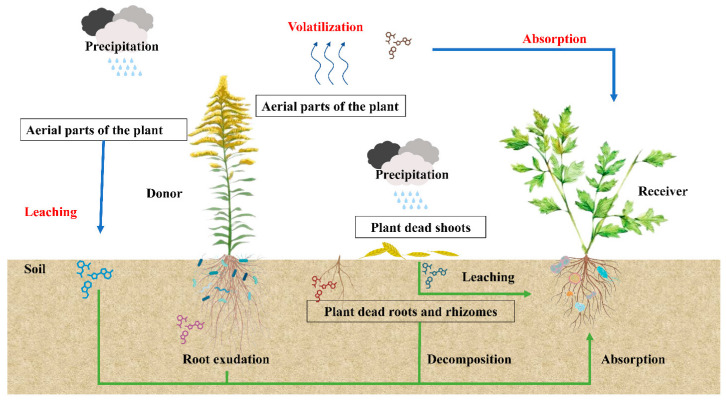
Illustrates the transfer of allelopathic compounds from a donor plant to a receiver plant through various pathways, including leaching, volatilization, root exudation, and decomposition. Compounds are released into the soil via precipitation or root exudation, volatilized into the atmosphere, or deposited through decomposing plant material, where they can be absorbed by neighboring plants. This chemical exchange plays an important role in shaping plant interactions, influencing competitive dynamics and ecosystem structure.

Certain plant parts manufacture allelochemicals, which are then released into the environment around the plants through leachates from rainfall, volatilization from the plants, exudation of roots, or the breakdown of plant debris and litter [100]. Allelochemicals, the byproducts of secondary metabolism, are found in every part of the plant, including the leaves, stems, flowers, seeds, fruits, and/or roots. There are various ways in which the producing plant can release these products: plant waste volatilization, foliar leaching, root exudations, and decomposition (Figure 4). VOCs are widely distributed plant allelochemicals and secondary metabolites that plants volatilize [147]. Mevalonic acid (MVA), methylerythritol phosphate (MEP), lipoxygenase (LOX), and shikimate/phenylalanine are the four basic mechanisms for the production of volatile organic compounds (VOCs). Terpenoids, phenylpropanoids/benzenoids, and fatty acid derivatives are among the VOCs that plants can create and release [148]. According to [149], these plants release volatile organic compounds (VOCs) that serve a variety of ecological purposes, including chemical communication, kin recognition, insect attraction or repulsion, and many more. While most studies on volatile organic compounds (VOCs) in plants focus on aboveground chemical signals, an increasing body of research indicates that VOCs are also crucial for belowground plant-plant interactions [150]. Volatile oil of *C. odoratum* at 800 mg/L inhibits the growth of *Pyricularia grisea*, *Phytophthora nicotianae*, and *Fusarium axysporum*.

Table 3 summarizes studies during the last decade reporting VOC-mediated allelopathic effects of invasive plants. According to these findings, invading species may have a significant negative impact on native plants’ chemical habitats by releasing volatile allelochemicals into the environment that prevent or lessen native species’ ability to germinate and flourish. Allelopathic substances can accomplish this by lowering the photosynthetic efficiency of recipient plants or interfering with the mechanisms involved in cell division (mitosis) [53]. The release of allelochemicals appears to be the fundamental mechanism, causing the receiver to emit reactive oxygen species, which set off a chain of signals and ultimately alter gene expression across the entire genome [151]. To clarify the mechanism(s) of action of volatile allelochemicals, more research is required. Litter is another way that volatile allelochemicals from invasive species might enter the rhizosphere. It is well known that litter volatile chemicals are tenacious and can be found years after litter deposition [152]. A pioneering study outside the scope of this review [27] indicates that an invasive species’ ability to spread is greatly influenced by volatile chemicals found in its litter. We therefore recommend further research to ascertain the allelopathic potential of the volatile organic compounds (VOCs) that invasive plants leave behind on native species and the length of time that residues remain bioactive after invasive plants have been removed.

### 3.2. Herbivory and Invasive Plant Species Interactions

#### 3.2.1. Insects

**Figure 5:**
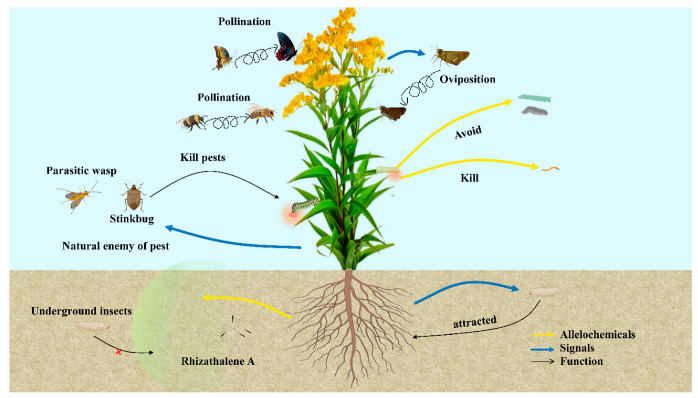
Role of secondary metabolites in plant–insect interactions.

Invasive plant species have a major impact on the insect ecosystem due to the actions of secondary metabolites. These compounds perform different roles in influencing insect ecology, behavior, and community dynamics. Secondary metabolites have defensive functions. For example, invasive plant species produce phytochemical compounds that work as feeding deterrents and stimulants for herbivores, influencing their choice of host and foraging [157]. Invasive plant compounds change the nutritional quality, affecting herbivore performance and fitness [158]. These secondary metabolites also affect the interactions between invasive plants and their mutualistic or antagonistic insect companions, including pollinators or natural enemies [159,160]. Actually, invasive plants use these secondary metabolites as defense strategies against herbivory. This may include direct avoidance of feeding or toxicity to herbivores [158], as shown in Figure 5. These might change in the abundance and distribution of insects and modification in the tropic relationships that exist within the ecosystem. The chemical signals facilitated by secondary metabolites affect the mutualistic relationship between the herbivores, pollinators, or predators and invasive plant species, which may affect the reproductive rate and spread the invasive plant population [146]. In order to improve management and conservation strategies for invasive species, researchers can gain a better understanding of the mechanisms underlying the success of invasive plants and their ecological impacts on insect communities by clarifying the roles that secondary metabolites play in mediating plant-insect interactions in natural settings. Table 4 lists the invasive plant species utilized to control insect infestations. Cotton aphids are poisoned by *E. adenophorum* chloroform extracts. The primary allelochemicals found to be harmful were *Aphis gossypii* and *eupatorin A*. Within 48 h, *eupatorin A* at 2 mg/mL can eliminate 81% of cotton aphids [161,162]. This compound also inhibits the enzymatic activity of AChE and NaK-ATPase of the cotton aphids in vitro and in vivo. A-1, P-1, Zi-2, and the leachates of *E. adenophorum* had anti-feeding activity to the fourth instars of *Pieris rapae,* as reported by [163]. The weed extracts have strong insecticidal activity against four stored grain insects: rice weevil, maize weevil, Chinese bean weevil, and European bean weevil [164]. Epifriedelinol, stigmasterol, octacosanoic acid, 8-daucos tero1, 2-isopropeny1-5-acetyl-6-hydrxybenzofuran aceate, and o-hydroxy einnamic acid were isolated from the *E. adenophorum* [165]. The ethanolic extract from *Alternanthera brasiliana* (L.) Kuntze’s leaves was evaluated for its insecticidal activity against the Hamburg strain of *Drosophila melanogaster* [166]. After exposed for 24 to 48 h, researchers discovered that the ethanolic extract at the studied quantities had a slight insecticidal impact. Phytomolecules like kaempferol and kaempferol analogs [167], quercetin and quercetin analogs [168], stigmasterol [169], β-sitosterol [170], spinasterol [171], and ferulic acid [172], which were isolated earlier from *Alternanthera brasiliana* (L.) Kuntze, might be in charge of this insecticidal characteristic. When *A. adenophora* was extracted in methanol, it showed a significant toxic effect against mites, *Sarcoptes scabiei* and *Psoroptes cuniculi* [173].

When the leaves of *A. adenophora* were extracted in ethyl acetate, the compounds were identified using gas chromatography-mass spectrometry. 5,6-dihydroxycadinan-3-ene-2,7-dione was found to be most effective against *Meloidogyne incognita* [174]. The essential oils called precocenes from A. conyzoides affect the digestive system, and the anti-juvenile hormones of the oil caused abnormalities in metamorphosis [175]. According to [176], the oil extracts also exhibited genotypic or phenotypic abnormalities in the immature *Aedes, Anopheles*, and *Culex* species. Also, the secondary metabolites in this invasive plant showed promising results against many insect pests, such as *Helicoverpa armigera*, *Phytophthora megakarya, Rhipicephalus microplus, Tribolium castaneum, Diaphania hyalinata,* and *Plutella xylostella* [177]. The methanolic extracts of *M. micrantha* significantly repelled Oriental fruit flies in the field [135,178]. According to [179], *Plutella xylostella, Phyllotretast riolata*, and *Phaedon brassicae* showed oviposition deterrence when volatile oils of *M. micrantha* were used at a dose of 5–10 µL/plant. The antecedent effects of crude extract of *M. micrantha* on l–2 instars of *Pieris rapae* and 2–3 instars of *Plutella xylostella* were 80% and 70%, respectively [180]. Allelochemicals such as mikanin, eupalitin, eupafolin, (3,4′,5,7-tetra-hydroxy 6- methoxyflavone 3-O-β-D-glucopyranoside, luteolin, 3,5-di-O-caffeoylquinic acid n-butyl ester, and 3,4-di-O-caffeoylquinic acid n-butyl ester were identified from *M. micrantha* [105]. β-cubebene, terpinolene, β-caryophyllene, 1imonene, β-farnesene, ocimene, δcadino1, γ-terpinene, ethylnaphthalene, a-caryophy11ene, β-cadinene + isocaryophyllene, δ-bisabolene, and β-bisabolene+cubebo1 were determined as the main compounds in essential oil of *M. micrantha* [180]. The volatile oil of *Chromolaena odoratum* is a strong oviposition deterrent of striped flea beetle (*Phyllotreta striolata*) and diamondback moth (DBM) (*Plutella xylostella*) at dose of 10–20 µL/plant [181]. The alcohol extract and its chloroform fraction exhibited strong repellent effects (80%) against DBM [182]. The alcohol extracts of *C. odoratum* effectively deterred the oviposition of DBM, and the active compounds were identified as chalcones and flavonols [183].

**Table 4:** List of invasive plants used against Insects.

Invasive Plant	Extract	Phytochemical	Target Insect	Mode of Action	References
*Ageratina adenophora* (Spreng).	Aqueous	Epifriedelinol, stigmasterol, octacosanoic acid, 8-daucos tero1, 2- isopropeny1-5- acetyl-6-hydrxybenzofuran aceate and o-hydroxy einnamic acid	Rice weevil, maize weevil, Chinese bean weevil and European bean weevil	Toxicity	[161]
*Alternanthera brasiliana* (L.) Kuntze	Ethanolic extract	Kaempferol and kaempferol analogs, quercetin and quercetin analogs, stigmasterol, β-sitosterol, spinasterol and ferulic acid	*Drosophila melanogaster*	Toxicity	[184]
*Ageratina adenophora* (Spreng).	Ethyl acetate	Cadinene sesquiterpenes, 5,6-dihydroxycadinan-3-ene-2,7-dione	*Meloidogyne incognita*	Antinemic activity	[174]
*Ageratum conyzoides* L. Lemmon grass	Crude extracts	PONNEEM	*Aedes, Anopheles, Culex* spp.	Affects the oviposition rate and increases the deterrence percentage	[176,185]
	Methanol extracts	6-demethyoxyageratochromene (precocene I) and ageratochromene (precocene II)	*Preris rapae* and *Plutella xyloaella*	Antifeeding effects	[125]
*Mikania micrantha* Kunth.	Methonal extract	Mikanin, eupalitin, eupafolin, (3,4′,5,7-tetra-hydroxy 6- methoxyflavone 3-O-β-D-glucopyranoside, luteolin, 3,5-di-O-caffeoylquinic acid n-butyl ester and 3,4-di-O-caffeoylquinic acid n-butyl ester were identified from M. micrantha	Oriental fruit fly	Repellent effects	[105]
	EOs	β-cubebene, terpinolene, β-caryophyllene, 1imonene, β-farnesene, ocimene, δcadino1, γ-terpinene, ethylnaphthalene, a-caryophy11ene,	*Plutella xylostella, Phyllotretast riolata* and *Phaedon brassicae*	Oviposition deterrent	[179,180]
*Chromolaena* *odoratum* L.	Alcohol extracts	Chalcones and flavonols	*Plutella xylostella*	Repellent	[183]
	Crude extracts		*Helicoverpa armigera*	Antifeeding effects	[181]
	EOs	Trans-caryophyllene, β-cadinene, a-copaene, caryophyllene oxide, germacrene-D and n-humuhne	*Phyllotreta striolata*	Oviposition deterrent	[186]
*Parthenium hysterophorus* L.	Flower, leaf stem powders	Parthenin ageratochromene, precocene I, and precocene II have strong insecticidal effects, endo-borneol, farnesol, quercetin, kaempferol, and its glucosides	*Callosobruchus chinensis*	Repellency, inhibit cholinesterase	[187]
	Aqueous leaf and stem		*Aedes aegypti, Sitophious oryzae*	Toxic and oviposition deterrent	[188,189]
*Melia azedarach* L.	Aqueous extract Fruits	Azadirachtin	*Callosobruchus maculatus*	Toxicity and repellency	[190]

#### 3.2.2. Soil Microorganism

Invasive plant species often produce secondary metabolites—organic compounds not directly involved in the primary metabolic processes of growth, development, and reproduction but play important roles in interactions with other organisms. These secondary metabolites not only modulate soil microbial populations but also defend against herbivores, pathogens, and allelopathy. Invasive plant species, plant-soil microbes, and secondary metabolites, there is a complex interaction among them, which impacts ecosystem dynamics. When invasive plant species invade an area, the interaction between the native below-ground and above-ground plant species changes. These interactions affect not only the structure of soil inputs derived from plants but also the quality, quantity, and timing [191]. In this situation, invasive plant species invasion may change the timing of litter formation, types, quantities, and the nutrients that are absorbed by the soil. Sometimes, the frequency and intensity of fire increase due to the increased litter production from some invasive plant species [192]. When invasive plants excrete unknown exudates (roots of plant species exude secondary metabolites), they may change the composition and role of the soil community [193]. Invasive plant species produce allelochemicals from the roots, primarily affecting plant-to-plant interactions, which describes the success of invasive plants [194]. However, recently studies suggest that allelochemicals change the interaction between native plant species and soil ecosystems. For example, in Western North America, *Centaurea diffusa* spreads extensively in its environment, with the roots of *C. diffusa* releasing the allelochemicals 8-hydroxyquinoline, which functions as an anti-bacterial agent [195]. According to [196], *C. diffusa* alters the soil microbial community due to these allelochemicals. Invasive plant species also release some novel chemicals which can change the soil microbial community. There is a diverse research gap in this case. For example, in Hawaii, *Myrica faya* invaded nitrogen-limited areas along with its nitrogen-fixing root symbionts (*Frankia* spp.), affecting nitrogen cycling and changing the composition of the plant community [197]. Invasive plant species directly change the physical properties of the soil environment, initiating ecosystem modifications that lead to control of soil functions and composition. For example, in the western United States, *Halogeton glomeratus,* a plant species that invades rangeland, accumulates sodium from the below soil to its biomass. In the invaded soils, this invasive plant species increases the sodium concentrations, which creates problems and modifications in microbial communities [197]. Invasive plant species produce some chemicals in the soil that prevent the growth of nearby plants and microbes. The compositions and activity of soil microbial communities changed due to the ability of allelochemicals, which selectively promote the growth of some microbial taxa while inhibiting others [28]. Nitrogen-fixing bacteria or mycorrhizal fungi might be prevented from growing by the phenolic compounds from invasive plant species, which alter the plant and microbial interactions and the mechanisms involved in the cycling of nutrients [36]. Also, Ref. [198] studied that some invasive plant species produced allelochemicals that affected the rhizosphere microbiome by serving as nutrient sources or signaling molecules for specific microbial populations. Some invasive plant species produced flavonoids, which change the functions and structure of the soil microbial community. The growth of rhizobacteria is influenced by these flavonoids, which promote the growth of plants or suppress diseases [199]. Some invasive plant species directly inhibit the growth of soil-borne pathogens or competing microorganisms through secondary metabolites with antimicrobial properties [73]. The presence or activity of harmful microbes is decreased by the invasive plant species, allowing them to outcompete the native plants, further exacerbating their impact on ecosystem structure and function. Table 5 summarizes the impacts of invasive plant species on native soil microbe communities.

### 3.3. Arbuscular Mycorrhizal Fungi (AMF)

Arbuscular mycorrhizal fungi (AMF) form symbiotic relationships with a diverse range of plant species and are important and prevalent soil microorganisms in terrestrial environments [205]. The competition of invasive plant species is influenced by AMF [206]. The invasive plant species *Centaurea maculosa* in North America utilize the mycorrhizal network that connects the roots of native plants [137]. In a new environment, an invasive plant, *S. canadensis,* in China has the ability to change AMF composition, making it more successful in invaded areas than native plant species [207]. Invasive plant species disrupt the symbiotic relationship between native plant species and AMF through secondary metabolites. For example, the invasive plant *Alliaria petiolate* cannot form symbiosis with AMF due to the secondary metabolites, which cannot develop symbiosis with AMF [208]. The specific flavonoids from *A. petiolate* cause invaded soil to have a significantly larger inhibitory action on AMF compared to its native soil [209]. Furthermore, secondary compounds derived from *A. petiolata* change the AMF community linked to native sugar maple seedlings and inhibit AMF hyphal and spore germination [210], despite abundance research showing invasive plants can disrupt local hosts’ AMF symbioses through secondary metabolites [207].

#### 3.3.1. Symbiotic Relationship Between Invasive/Native Plants and AMF Communities

Plant and fungal metabolites mediate the plant–AMF symbiosis. Primary, specific, and phytohormone metabolites facilitate partner recognition, colonization, and the development of a symbiotic relationship in the plant–AMF symbiosis. Root-released quercetin and 2-hydroxy fatty acids trigger compound-specific morphological AM fungal responses during pre-symbiotic communication. Next, hyphopodium formation on the root surface is triggered by strigolactone and cutin monomers (1,16-hexadecanediol and 16-hydroxyhexadecanoic acid). It is known that the pre-symbiotic phase of the association between AMF colonies and plants determines the specificity of such a relationship [211,212]. Phytohormones play an important role between AMF and plant species as a signaling molecule. According to [212,213], strigolactones, auxins, abscisic acid, brassinosteroids, and gibberellic acid are involved in their function from the first detection of AMF in the soil to the ultimate development of mycorrhiza. Auxin is necessary for both the early stage of fungal development and the differentiation of arbuscules, while the synthesis of arbuscules is controlled by gibberellic acids [213]. During the early colonization stage, plant–AMF interactions are facilitated by carotenoid pathways and control of signaling. Phytohormones boost gibberellic acid production by varying the ratio of the salicylic acid and jasmonate signaling pathways and affect plant immunity. Through the symbiotic relationship, plants produce maximum sugar and metabolites for the tricarboxylic acid cycle, boosting photosynthetic sharing. Changes in the primary metabolites also influence arbuscular mycorrhiza growth, affecting the synthesis of specialized metabolites [214]. AM has a beneficial impact on the synthesis of specialized metabolites by boosting metabolite biosynthesis pathways or increasing plant biomass. According to [215], changing plant immunity through plant hormones enables mycorrhizal fungi to influence key plant chemicals that deter pests. For example, Senecio genus plants produce pyrrolizidine alkaloids, their primary defense compounds, in response to AMF invasion.

#### 3.3.2. Mechanisms by Which Invasive Plants Affect Native Plant Mycorrhizal Fungi

##### Ecological Mechanisms

**Figure 6:**
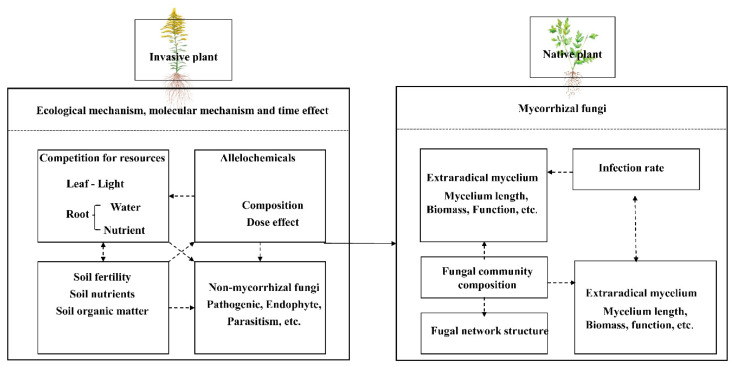
Conceptual illustration showing how invasive plants affect the symbiotic mycorrhizal fungi in native plant roots Solid arrows indicate change; dotted arrows indicate possible relationships.

Invasive plants can affect native plants through a variety of ecological mechanisms. At present, relevant research mainly focuses on species competition, soil nutrient changes, and allelopathy. As shown in Table 6, invasive plants tend to compete for resources (light and nutrients, etc.). For example, *Solanum carolinense* has a strong ability to reproduce asexually and can spread rapidly [216]. Invasive plants can also inhibit photosynthesis in native plants; reduced photosynthates may inhibit native plant mycorrhizal fungal infection [217] (Figure 6). Invasive plants tend to have strong nutrient competitiveness and a large amount of root exudates [218]. Invasive plants can also improve and enhance soil nutrients through litter and root exudates, forming a positive feedback loop between plant and soil [219]. This process reduces the infection rate of mycorrhizal fungi in native plants (such as *Solidago decurrens* and *Andropogon gerardii*) and alters the community composition of mycorrhizal fungi [220] (Figure 6). Invasive plants can also direct affect native plant mycorrhizal fungi through allelopathy [221] (Figure 6). Allelopathic substances produced by invasive plants (such as flavonoids and glucosides, etc.) inhibit the germination, growth, and infection of mycorrhizal fungi spores, thereby inhibiting the growth of native plants dependent on these fungi [222]. These allelopathic substances may also inhibit mycorrhizal fungal infection by inhibiting the growth of native plants, thereby reducing the carbon supply of plants to mycorrhizal fungi [223]. In addition, allelopathic substances secreted by foreign plants may also affect the non-mycorrhizal fungi of native plants, thereby indirectly affecting the mycorrhizal fungi of native plants (Figure 6). In fact, changes in native plant mycorrhizal fungi may result from a combination of mechanisms [224]. The influence of different invasive plants on native plant mycorrhizal fungi may be different, and the response of native plant mycorrhizal fungi to each mechanism may also be different, resulting in varied trends in the influence of foreign plant invasion on different native mycorrhizal fungi.

##### Molecular Mechanism

**Figure 7:**
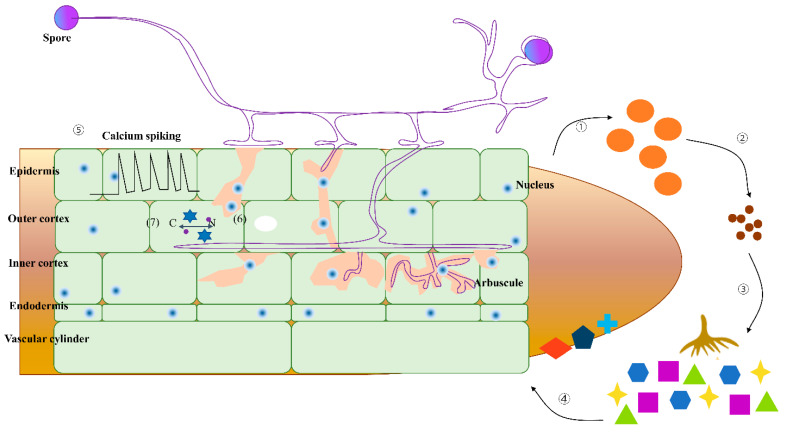
Conceptual framework illustrating the symbiotic relationship between plants and mycorrhizal fungi (adapted from Martin et al [236] Roots secrete signaling molecules (1) that enhance AMF spore germinations (2) and mycelium branching in the soil. AMF secretes mycorrhizal factors (3), which are recognized by receptor proteins in root cells (4) and then stimulate the calcium signaling pathway (5) to pledgee creation of invasion lines of the mycorrhizal fungi (6). After that, nutrient and carbon exchange between mycorrhizae also requires a series of enzymes and transport proteins at the root–mycorrhiza interface (7).

The symbiotic relationship between plants and mycorrhizal fungi is a complex signal transduction process. In the creation of arbuscular mycorrhizal fungi, signal exchange between the root system and AMF is the first step [231]. According to [232], strigolactones are signaling molecules secreted by the root system (e.g., *Lotus japonicus*) that not only encourage the AMF spore germination but also enhance the chance of hyphae contact with the root system. Mycorrhizal fungi secreted an array of signaling molecules (i.e., mycorrhizal factors, like LCOs, CO4/CO5, and PsMiSSP10b). As shown in Figure 7, corresponding receptors in the root system recognize these molecules and activate the calcium ion signaling pathway, which in turn produces an infection line and initiates the mycorrhizal fungi’s infection process [233]. For example, short chitosan oligosaccharide (CO4/CO5) and lipochitosaccharide (LCOs) secreted by rice AMF are recognized by heteromers of LysM receptor-like kinases in rice, including OsMYR1/OsLYK2 and OsCERK1 [234]. LCOs and CO4/CO5 factors released by AMF are recognized by NFR1/LYK3H in leguminous plants [235]. Mycorrhizal fungi also produce plant cell wall decomposition enzymes (PCWDEs) by producing small secreted proteins (MiSSPs) to recognize hosts [127], disrupting the cell wall of the host plant and removing the “barrier” to the establishment of symbiotic relationships. The important relationship between the mycorrhizal fungi and roots lies in the exchange of nutrients and carbon (lipids and sugars) [234], which comprises a sequence of lipid synthases and nutrient transporters. Mitogen-activated protein kinases (MAPKs) are key pillars in AMF and plant signaling. According to [236], it is the STR transporters that carry fatty acids through alfalfa *Medicago truncatula*. Invasive plant species inhibit native plant mycorrhizal fungal infections by changing these transport enzymes, ultimately impacting native plant growth. For example, lipid synthase (FatM and RAM2) mutations in alfalfa roots, which control the supply of lipids needed by mycorrhizal fungi in plants, were found to reduce the infection rate of AMF [236].

## 4. Management of Invasive Plant Species

In order to manage the invasive plant species, we should focus on the secondary metabolites responsible for their invasiveness. Here are a few methods to elaborate specific secondary metabolites that significantly enhance the plant species’ invasiveness. According to [237], there should be a focus on plant species which exhibit allelopathic effects or competitive advantages. Biological control refers to managing invasive plant species by introducing another living organism, such as insects, diseases, or herbivores, to prevent invasive plant species from spreading and growing. Introducing natural enemies from the exotic plant species’ habitat can reduce the growth and reproduction of specific plant species. Weed scientists and entomologists carefully select those natural enemies from exotic habitats to ensure they target only the invasive plant species without causing harm to other living organisms. According to [238], biological control of invasive weeds offers environmentally friendly and sustainable strategies through careful observation and adaptive management. To reduce the impact of invasive plant species, biological control is often combined with other control techniques. To stop the production or release of harmful secondary metabolites from invasive plant species, targeted chemical management strategies are needed [239]. To reduce the effectiveness of secondary metabolites of invasive plant species, land management strategies can be used to change the soil properties and microbial population [237]. Reducing soil-borne allelopathic chemicals’ effects use cover crops or targeted plant techniques [240]. Developing native cultivar plant species through genetic modification or selective breeding can enhance tolerance or resistance to secondary metabolites of invasive plant species. Additionally, exploring options to modify invasive plants to produce fewer secondary metabolites that confer invasiveness may be beneficial [241].

To reduce the impact of invasive plant species’ secondary metabolites in invaded ecosystems, integrated pest management (IPM) strategies should be used to decrease dependence on a single control method and enhance the diversity of native plant species. While taking the ecological environment into consideration, IPM approaches combine different control methods [242]. According to [243], IPM concentrates on identifying invasive plant species and their secondary metabolites in newly invaded area. Before invasive plant species establish dominance, IPM strategy can manage them effectively. Educating stakeholders, land managers, and the general public regarding stopping the introduction and spread of invasive plant species, as well as selecting appropriate plant species for gardening and landscaping, is essential [242]. Efficient and quick management strategies for invasive plant species should incorporate multiple tactics to minimize their negative effects on ecosystems. A good management strategy integrates methods for early detection and quick response. Strict biosecurity protocols and public awareness campaigns can prevent the introduction and spread of invasive plant species. Early detection and timely identification enable immediate actions to prevent the invasive plant species from growing and spreading. Management strategies may include biological control through the introduction of natural enemies, mechanical control (e.g., manual removal or mowing), chemical control with weedicides, culture control through vegetation with native plant species, and microbial control. Implementing a combination of these available techniques can open new avenues for managing invasive plant species, preserving biodiversity, and promoting ecosystem health. Regular monitoring to evaluate the efficacy of these measures is also crucial.

## 5. Conclusions and Future Perspectives

Plant invasions highlight the significant impact of non-native species on ecosystems, biodiversity, and human activities. Understanding the mechanisms of plant invasions is essential for effective management and conservation efforts. Secondary metabolites in invasive plant species play a pivotal role in facilitating plant invasions by influencing various stages of the invasion process. Phenolic compounds, alkaloids, terpeniods, and other phytochemicals are important volatile organic compounds that serve as major contributors to the success of invasive plants. Invasive plants outcompete native ones due to their diverse chemical properties, alter soil composition, and modify ecological interactions. Due to the presence of secondary metabolites, invasive plants possess chemical defense mechanisms against herbivores, pathogens, and competing vegetation. The complex functions of these compounds not only support the establishment and spread of invasive species but also impact ecosystem dynamics and functions. In the future, biological control methods for invasive plant species will involve harnessing living organisms such as insects, pathogens, or herbivores to effectively manage the proliferation and spread of invasive plant species. In the context of secondary metabolites and plant invasion, future research will likely focus on clarifying the molecular process controlling the production, release, and ecological functions of these compounds. Understanding secondary metabolites in plant invasion is pivotal for shaping the dynamics of invasive species and their interactions with native flora and fauna. By elucidating the mechanisms underlying the production and function of these metabolites, we can develop targeted management strategies to mitigate the impact of invasive plants on ecosystems. Future developments in omics technology will offer deeper insights into the intricate interactions between secondary metabolites and plant invasion, opening the door for more successful conservation and management initiatives. These insights will be paired with ecological modeling and field research.

## Figures and Tables

**Table 1:** Secondary metabolites in invasive plant species and their mechanism.

Plant Species	Category	Compounds	Mechanism	References
*Artemisia tridentata* Nutt.	Volatile organic compounds	Methyl jasmonate	Activates expression of defense genes	[70]
*Alliaria petiolate* (M.Bieb.) Cavara & Grande	Phenolic compounds	Glucosinolates (sinigrin)	Mycorrhiza are suppressed by sinigrin, which breaks their mutualistic relationships with native plants.	[73]
*Ageratum conyzoides* L.		P-coumaric acid, gallic acid, ferulic acid, p-hydroxybenzoic acid, and anisic aci	Rice growth was adversely influenced by phytotoxins released into the soil rhizosphere by *A. conyzoides* residues and root exudates.	[74]
*Cymbopogon nardus* (L.) Rendle		N-Octanoyl tyramine	Inhibits ripening of *Lepidium sativum*, *L. sativa, Echinochloa crusgalli, Lolium multiflorum*	[49]
*Juglans nigra* L.		Juglone	Inhibitor of the essential enzyme for the formation of plastoquinone, hydroxyphenylpyruvate dioxygenase (HPPD), as well as other plants’ photosynthetic and respiratory electron transport systems	[47]
*Secale cereale* L.	Alkaloid compounds	Benzoxazinoid	Boosts benzoxazinoids’ synthesis and exudation from roots in reaction to nearby plants	[75]
*Echium plantagineum* L.		Pyrrolidine and Naphthoquinones	Provide a competitive edge over weeds and protect against livestock and insect herbivory.	[75]
*Senecio jacobaea* L.		Pyrrolizidine	Increased alkaloids produced in non-native range compared to native range; protection against generalists	[75]
*Imperata cylindrica* (L.) P. Beauv.	Tarpenes	Tabanone, 4-(2-butenylidene)-3,5,5-trimethyl-2-cyclohexen-1-one; cogongrass,	Impeded the growth of the garden onion’s roots, the lesser duckweed’s frond area, and the garden lettuce’s fresh weight gain.	[76]

**Table 2:** Biological properties of invasive plant species and their allelopathic effect.

Invasive Plant Species	Allelochemicals	Mode of Action	Effected Plants	References
*Solidago canadensis* L.	Kaempferol-3-O-d-glucoside	Growth	*Arabidopsis thaliana* (L.) Heynh., *Echinochloa colona* L.	[93]
*Ageratina adenophora* (Spreng).	Propan-2-ylidene (4,7-dimethyl-1-) tetrahydronaphthalene-1,4,4a, 8a, 2(1H, 7H) DTD and 6-hydroxy-5-isopropyl-3	Growth and development	*Osbeckia stellate buch.* HAM. EX D. DON and *Elsholtzia blanda* Benth.) Benth.	[109]
*Polygonum* cuspidatum Sieb. et Zucc	(−)-catechin, (−)-epicatechin, resveratroloside, and piceatannol	Growth	*Lepidium sativum* L.	[110]
*Chromolaena odoratum* L.	Globulol, α-cadinal, 1-hexadecanol, caryophyllene, (−)-spathulenol, and caryphyllene oxide hexadecane	Growth	*Eleusine indica* (L.) Gaertn, *Cyperus iria* L., and *Ageratum conyzoides* L.	[111]
*Ambrosia artemisiifolia* L.	α-pinent, β-pinene, cineole, camphene, spanthueol	Germinations and root growth	*Zea mays* L. (Corn)*, Triticum aestivum* L. and *Oryza sativa* L.	[112]
*Ageratum conyzoides* L.	Precocenes, sesquiterpenes, Gallic acid, proteocatechins acid and coumaric acid,	Germination up to 89%	*Parthenium hysterophorus* L.	[113]
*Conyza bonariensis* (L.) Cronquist	(4Z)-lachnophyllum lactone	Suppression of growth	*Cuscuta campestris* L.	[114]
*Eucalyptus camaldulensis* Dehnh.	Syringic acid, vanillic acid, gentisic, gallic, p-coumaric, p-hydroxybenzoic, and catechol	Suppression of germination and growth	*Portulaca oleracea* L.	[46]
*Eichhornia colona* L.	Tricin	Inhibit germination and seedling growth	*Glycine max* L. and *Oryzae sativa* L.	[16]
*Eucalyptus globulus* Labill.	Kaempferol 3-O-glucoside, hyperoside, and shikimic-succinic acids	Inhibit germination, growth and physiological parameters	*Agrostis stolonifera* L.	[115]
*Mikania micrantha* Kunth.	Dihydromikanolide, deoxymikanolide, 2,3-epoxy-1-hydroxy4,9-germacradiene12, 8:15,6-diolide.	Limit the length of the radicle and shoot.	*Trifolium repens* L., *Raphanus sativus* L., and *Lolium perenne* L.	[104]
*Parthenium hysterophorus* L.	Caffeic acid, parthenin	Suppress the growth of seedlings and germination	*Digitaria sanguinalis* (L.) Scop. and *Eleusine indica* (L.) Gaertn	[116]
*Asystasia gangetica* L.	(6R,9S)-3-oxo-α-ionol and indole-3-carboxaldehyde	Cause 10% yield reduction	*Cucumis sativus* L.	[117]
*Artemisia annuas* L.	Artemisinin	Prevent development and expansion of the roots	*Ipomoea lacunose* L., *Lactuca sativa* L., *Portulaca oleracea* L.	[118]
*Bidens pilosa* L.	Terpenes, phenolic acids, polyacetylenes, flavonoids, and fatty acids	Inhibit the growth	*Zea mays* L., *Sorghum bicolor* (L.) Moench., *Lactuca sativa* L, and *Vigna radiate* (L.) R. Wilczek	[119]
*Brachiaria mutica* (Forssk.) Stapf	Tannin, saponin	Germination and growth suppression	*Mimosa pudica* L.	[120]
*Cyperus rotundus* L.	Quercetin, luteolin, chrysin, rutin, myricitrin, catechin, apigenin, and chlorogenic acid	Lowers yield by 93% and 86%	*Oryza sativa* L.	[121]
*Pueraria montana* (Lour.) Merr.	12(13)-dien-bisabolene, 7-carboxy-8-hydroxy-1(2), and (-)-hamanasic acid A	Germination and Growth	*Lactuca sativa* L. and *Raphanus sativa* L., *Bidens pilosa* L. and *Lolium perenne* L.	[122]
*Datura stramonium* L.	Tropane alkaloids, Scopolamine, Hyoscyamine	Germination and growth	*Tagetes minuta* L. and *Amaranthus hybridus* L.	[123]
*Juglans nigra* L.	Juglone	Herbicidal activities	*Sonchus arvensis* L., *Cirsium arvense* L, *Papaver rhoeas* L., *Lamium amplexicaule* L.	[124]

**Table 3:** Research has been performed in the last few decades on the adverse effects of VOCs from invasive plant species on recipient plants.

Invasive Plants Species	Negative Effect on Receiver Plant Species	Receiver Plants Species	References
*Phytolacca americana*	Adverse effects on reproductive and morphological features	*Phytolacca acinosa*	[153]
*Prunus serotina*	Prevented the elongation of the roots, shoots, and germination	*Pinus sylvestris*	[154]
*Mikania micrantha*	Decreased rate of germination reduced levels of chlorophyll and reduced levels of malondialdehyde and reduced activity of superoxide dismutase	*Abutilon theophrasti, Bidens pilosa, Chrysanthemum coronarium* and *Lactuca sativa*	[155]
*Ageratina adenophora*	Reduced germination rate and limited height of seedlings reduced biomass of the shoots and roots	*Schima wallichii*	[132]
*Acacia longifolia*	Reduced biomass, shoot length, and root length	*Lolium multiflorum, Plantago lanceolata* and *Trifolium subterraneum*	[156]

**Table 5:** Summary of the impacts of invasive plant species on native soil microbe communities.

Invasive Plant Species	Novel Compounds	Impact on Soil Microbe	References
*Solidago gigantean* Aiton.	Sesquiterpene lactones	Affect soil microbial communities and inhibit microbial activity.	[151]
*Lantana camara* L.	Lantadene A	Disrupt microbial symbioses and alter soil microbial communities.	[200]
*Rubus armeniacus* Focke.	Ellagic acid	Allelopathic and antimicrobial effects on soil microbial populations.	[201]
*Centaurea maculosa* L.	Cnicin	Antifungal and antibacterial properties, affecting soil microbial composition.	[202]
*Alliaria petiolate* (M.Bieb.) Cavara & Grande	Glucosinolates (sinigrin)	Sinigrin suppresses mycorrhiza, therefore disrupting their mutualistic associations with native plants	[73]
*Phragmites australis* (Cav.) Trin. ex Steud.	Catechins	Influence microbial decomposition processes and soil nutrient cycling.	[203]
*Chromolaena odorata* L.	Acutellerin-40, 6,7-trimethy ether, 40, 5,6,7- tetramethoxyflavone, isosakuranetin	Greater amounts of flavonoids in the non-native range provide competitive advantages and better defense against soil borne pathogens	[204]

**Table 6:** Ecological mechanisms of invasive alien plant species.

Sr. No	Examples	Mechanism	References
1	*Parthenium hysterophorus* L., an invasive plant, may develop far more quickly than crops like *Sorghum bicolor* L. Moench) and *Zea mays* L.	Species competition	[20]
2	When 19 paired invasive and native plants in Hawaii were compared for resource usage efficiency, it was found that invasive plants had better rates of carbon absorption, light use, immediate nitrogen, and energy use.		[225]
3	Invasive plants have larger leaf nitrogen contents are less damaged by herbivores, according to comparisons between 47 paired invasive and non-invasive species’ leaf herbivore resistance and nutrient content.		[226]
4	When 125 invasive plants and 196 non-invasive plants are compared physiologically, that invasive plants are more advantageous in terms of growth rate, resource allocation, and stress resistance.		[227]
5	*Plantanum carolinense* L., *Solanum carolinense* L. is an exotic plant with great cold resistance and asexual reproduction.		[228]
6	*Solidago canadensis* L. is an invasive plant that can benefit from increasing nitrogen deposition and climate warming by acquiring more leaf resources.		[229]
7	Leachate of the invasive plant *Bothriochloa ischaemum* L. Keng prevents native species *Schizachyrium scoparium* (Michx.) Nash and *Andropogon gerardii* L. from germinating and growing	Allelochemicals	[230]
8	*Lactuca sativa* L., a native plant, seed germination and seedling growth inhibited by allelochemicals released by *S. canadensis* L. invasion		[155]
9	*Crystals of solanine* and oxalate are found in the exotic plant *Solanum carolinense* L.		[104]
10	To aid in its invasion, *P. hysterophorus* L. can release parthenin, vanillic acid, caffeic acid, and other allelochemicals		[88]

## Data Availability

All the data discussed in the article.
